# Radioembolization Practice in North America Versus Europe: Results from a Global Survey

**DOI:** 10.3390/curroncol33050285

**Published:** 2026-05-12

**Authors:** Grace Keane, Marnix G. E. H. Lam, Arthur J. A. T. Braat, Rutger C. G. Bruijnen, Nathalie Kaufmann, Hugo W. A. M. de Jong, Riad Salem, Maarten L. J. Smits

**Affiliations:** 1 Department of Radiology and Nuclear Medicine, University Medical Center Utrecht, 3584 CX Utrecht, The Netherlands; m.lam@umcutrecht.nl (M.G.E.H.L.);; 2 Boston Scientific Corporation, Marlborough, MA 01752, USA; 3 Department of Nuclear Medicine, Netherlands Cancer Institute, 1066 CX Amsterdam, The Netherlands; 4 Next Research, Contract Research Organization, 1010 Vienna, Austria; 5 Clinical Research, Cardiovascular and Interventional Radiological Society of Europe, 1010 Vienna, Austria; 6 Department of Radiology, Northwestern Feinberg School of Medicine, Chicago, IL 60611, USA

**Keywords:** radioembolization, global survey, interventional radiology, SPECT/CT, PET/CT, Yttrium-90, Holmium-166

## Abstract

Purpose: The Cardiovascular and Interventional Radiological Society of Europe (CIRSE) conducted an international survey on the real-life application of transarterial radioembolization (TARE). This sub-analysis of the complete survey evaluates intercontinental disparities in TARE practices. Materials and Methods: A survey of 32 multiple-choice questions was distributed to CIRSE members between November and December 2022. The questions addressed steps of the TARE workflow, including treatment work-up, planning and dosimetry, intervention, follow-up and innovations. Responses were curated to remove duplicates and incomplete entries and categorised into continental groups. Analysis focused on variations between Europe and North America and impacting factors in the respective regions were identified. Data is presented using descriptive statistics. Results: Responses were obtained from 30 countries and 133 hospitals, including 87 European and 21 North American centres. Hepatocellular carcinoma was the most common indication, constituting 61% of treatments in North America and 51% in Europe. North America predominantly used ^90^Y glass microspheres, whereas Europe used ^90^Y resin. Procedural differences included the adoption of intra-procedural CT imaging, utilized by all North American sites, versus 89% of European sites. Outpatient treatments were favoured in North America (85%), while in Europe, most patients remained hospitalized for one night (51%). Both regions increasingly emphasized dosimetry-guided treatments, with personalized dosimetry planning in 71% and 84% of North American and European sites, respectively. Conclusions: This North America–Europe comparison highlights regional differences in radioembolization practice between the leading continents in procedure volume, based on results of the CIRSE TARE survey. Specific intercontinental differences identified in this survey included hospitalization, product utilization, and procedural techniques.

## Simple Summary

The Cardiovascular and Interventional Radiological Society of Europe conducted an international survey on the real-life application of transarterial radioembolization. This sub-analysis of the complete survey evaluates intercontinental disparities in transarterial radioembolization practices. Key variations in the transarterial radioembolization workflow, including product utilisation, procedural techniques and hospitalisation management between Europe and North America, are addressed. By defining these regional differences, the study highlights how localised practices influence transarterial radioembolization application and how these findings may inform global harmonisation strategies.

## 1. Introduction

Radioembolization is an established treatment for primary and secondary liver cancers. Treatment involves the administration of beta-emitting microspheres of either yttrium-90 (^90^Y) or holmium-166 (^166^Ho) into the arterial hepatic vasculature. Early investigators demonstrated that by capitalising on the liver’s differential blood supply, the administration of microspheres within a tumour’s vascular network leads to a localised tumour dose and minimal dose to the surrounding healthy tissue. This results in effective tumour control, minimal toxicity and symptomatic relief across various hepatic malignancies [1,2,3,4].

Consequently, several radioembolization products were developed worldwide, including ^90^Y glass microspheres (TheraSphere, Boston Scientific, Marlborough, MA, USA), developed in Canada and available via humanitarian device exemption since 1999 [5], ^90^Y resin microspheres (SIR-Spheres, Sirtex Medical Ltd., Sydney, New South Wales, Australia), developed in Australia and CE marked since 2002, and ^166^Ho (Quiremspheres, Quirem Medical, Deventer, Overijssel, The Netherlands), developed in the Netherlands and CE marked since 2015 [6].

The evolution of radioembolization has seen procedural refinements (dose escalation guided by dosimetry planning [7,8] and selective microsphere administrations [9,10]), technical advancements (new scout agents [11] and catheter innovations [12]), and increased technology integration (the incorporation of intra-procedural CT imaging [13] and dosimetry software [14]). However, the adoption of these advancements varies geographically. Factors such as regulatory differences, product availability and reimbursement considerations shape global practice patterns. The relative impact of each of these factors is not well defined.

The aim of this study was to utilise the data of the previously published CIRSE Global Radioembolization Survey [15] to evaluate intercontinental disparities in radioembolization practices.

## 2. Materials and Methods

A comprehensive database of evidence on the real-life clinical application of this therapy was collated as part of a global survey by the Cardiovascular Interventional Radiology Society of Europe (CIRSE). The survey was developed jointly by the UMC Utrecht, CIRSE and Next Research (CIRSE’s contract research organization) and focused on key steps in the radioembolization workflow, including treatment work-up, treatment planning and dosimetry, intervention, follow-up and innovations. A full overview of the questions is available (Table 1). The time period investigated was 2017–2022. Implementation was via a web-based questionnaire, this was sent as an e-mail to all CIRSE members, local member societies in Europe and international sister societies. The survey was launched on 3 November 2022 and closed on 24 December 2022; three reminder e-mails were sent to delegates in the intervening period.

All entries in the web database were curated manually, which involved identifying and removing duplicates and incomplete responses from the dataset. Responses were categorized into continental groups, namely Europe, North America, South America, Asia, Oceania, and Africa. Since the majority of responses originated from European and North American sites, analysis was focused on discerning disparities between these two continents. Data analysis was performed using Python (3.9.7). Descriptive statistics were used to put results into context. Chi-square tests of homogeneity were applied selectively to limited endpoints. Due to the small sample size these analyses are exploratory.

## 3. Results

### 3.1. General Overview

**Figure 1:**
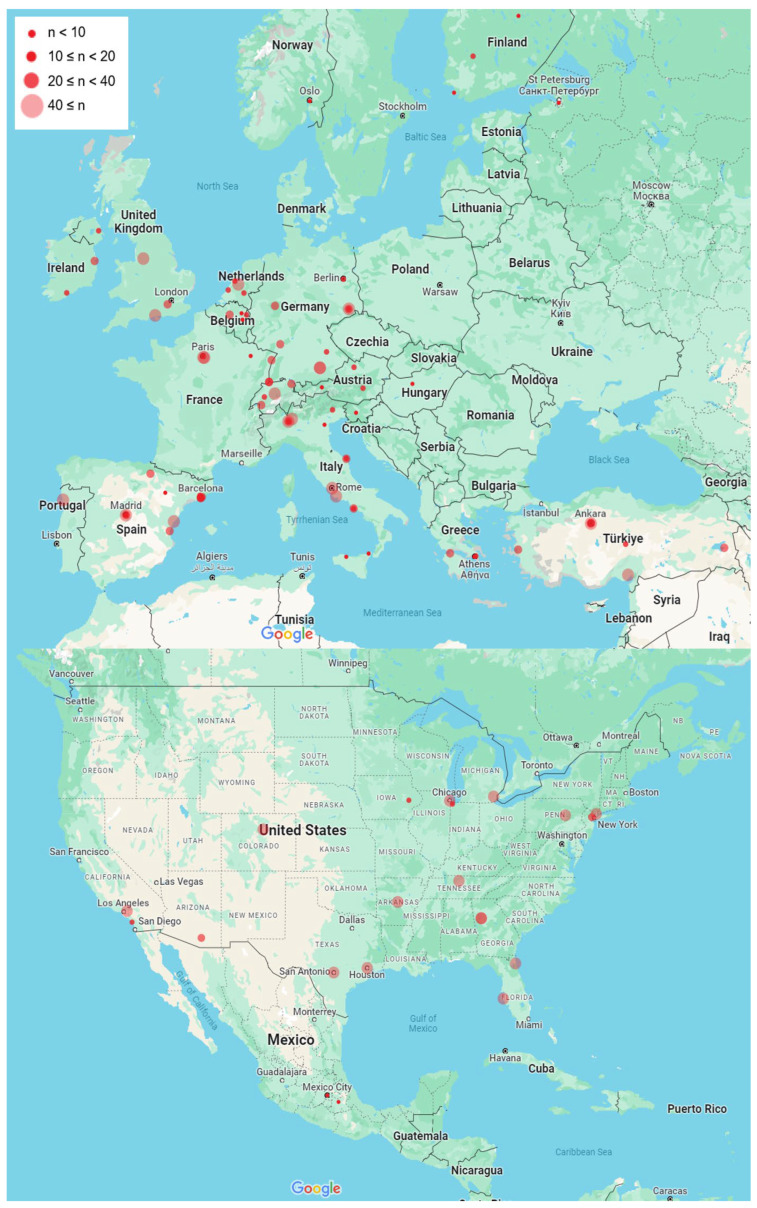
The distribution of responding centres in Europe and North America. The key indicates the number of procedures per centre in 2022. This research was initially published in CVIR by Keane et al. [15] (Copyright by CC BY 4.0.).

A total of 133 centres across 30 countries provided complete data for the survey. Of these, 18 countries were within Europe (*n* = 87 centres) and two were within North America (*n* = 21 centres) (Figure 1).

The average number of treatments performed per responding centre over all 5 years in North America was estimated to be more than double that in Europe (53 treatments per site in North Americas versus 20 in Europe). Consequently, among the responding sites, there were considerably more high-volume centres (i.e., centres performing at least one radioembolization per week) in North America (57%) than Europe (14%) (Table 2). In both continents, the majority of centres referred patients through a multidisciplinary team including interventional radiologists (Europe: 91%, North America: 86%).

Hepatocellular carcinoma was the most common indication (Europe: 51%, North America: 61%), followed by colorectal liver metastasis in Europe (19%) and cholangiocarcinoma in North America (15%) (Table 3).

**Figure 2:**
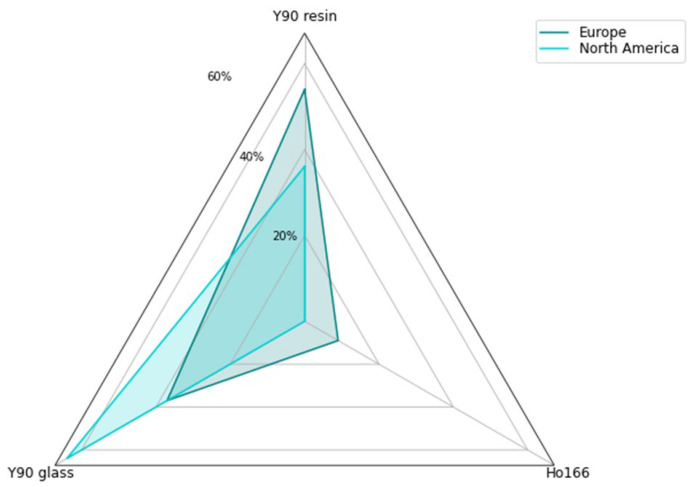
Percentage split of total procedures by microsphere product.

From 2017 to 2022, an estimated 10,680 procedures were completed by responding sites in Europe (54% ^90^Y resin, 37% ^90^Y glass, and 9% ^166^Ho), while in North America, 6725 procedures were performed by responding sites (64% ^90^Y glass and 36% ^90^Y resin) (*p* < 0.05) (Figure 2).

### 3.2. Pre-Treatment Work-Up

Prescription practices were generally consistent between continents, with similar rates of prophylactic antiemetics (Europe: 24%, North America: 21%) and proton pump inhibitors (both 21%). Discrepancies arose in the use of pain medication, notably opioids, prescribed in 7% of cases in Europe versus 15% in North America.

**Figure 3:**
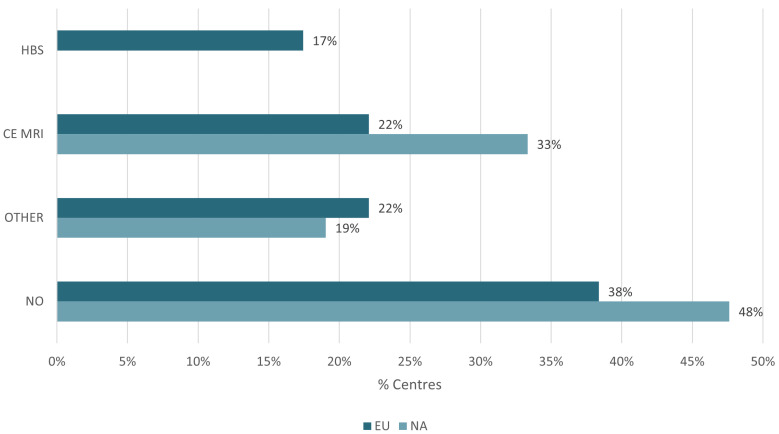
Percentage usage of image-based liver function assessment as part of the treatment work-up by region. HBS = hepatobiliary scintigraphy, CE MRI = contrast-enhanced MRI, Other = other form of image based liver function assessment, No = no form of image based liver function assessment.

In Europe, a higher proportion of responding centres (62%) conducted image-based liver function assessment compared to North America (52%). Methodological disparities were evident; for instance, hepatobiliary scintigraphy (HBS) was utilized by 17% of responding European centres, while no North American sites performed this. Instead, contrast-enhanced MRI (CE-MRI) with liver-specific agents dominated in North America (33%) (Figure 3).

### 3.3. Treatment Planning and Dosimetry

SPECT/CT was the preferred modality for pre-treatment scintigraphy in both regions. A clear continental disparity was evident in the perception of ^99m^Tc-MAA reliability for intrahepatic dosimetry. A total of 94% of European sites considered it reliable, compared with 6% that found it unreliable. In contrast, among North American sites, 81% rated it as reliable and 19% as unreliable (*p* = 0.05). (Table 4).

North American sites were less prone to exclude patients from treatment due to poor tumour ^99m^Tc-MAA-targeting; only 43% of centres would do so, in contrast to 64% of European centres. Despite uncertainty in the predictive capabilities of ^99m^Tc-MAA, 71% of users in North America implemented personalized dosimetry using scout scintigraphy, and 84% of users in Europe (*p* = 0.20). Medical Internal Radiation Dose (MIRD)-based multi-compartment dosimetry was the favoured dose calculation method in North America for both ^90^Y resin (29% of responders) and ^90^Y glass (48% of responders). MIRD multi-compartment dosimetry was also the preferred method in Europe for all products (^90^Y resin: 41%, ^90^Y glass: 31%, and ^166^Ho: 21%).

**Figure 4:**
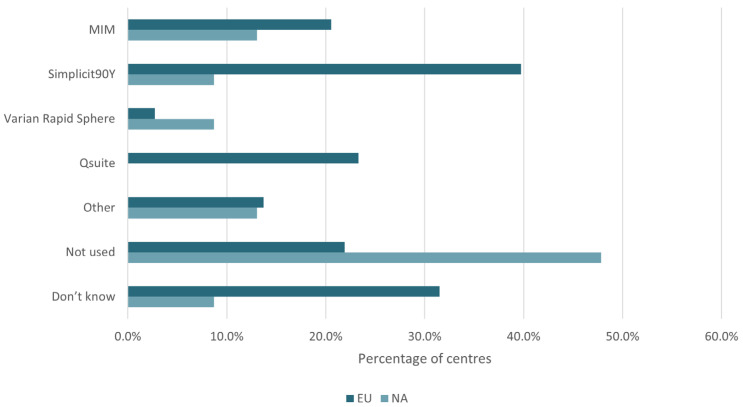
The percentage usage of dosimetry software by region.

**Figure 5:**
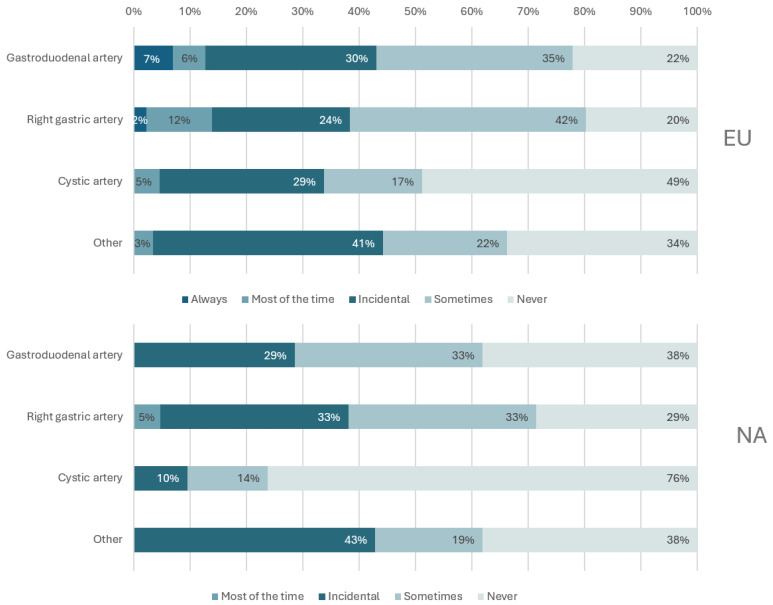
Coil embolization rates of the gastroduodenal, right gastric, cystic and other non-target vessels by region.

Dosimetry software was more widely adopted among responding sites in Europe (65%) than North America (44%). The most commonly used software packages in Europe and North America were Simplicit90YTM (Mirada Medical, Oxford, UK) and SurePlanTM MRT (MIM Software Inc., Beachwood, OH, USA) (Figure 4). In general, coil embolization of non-target vessels was rare; however, routine coil embolization was more prevalent in Europe than in North America. In North America, no sites reported always embolizing any of the considered vessels; in Europe, 7% of sites indicated they always embolized the gastroduodenal artery and 2% the right gastric artery (Figure 5). The intended vascular treatment or injection level (e.g., subsegmental, segmental, sectional, or lobar), a key determinant of coil embolization use, was not captured. The results should be interpreted in light of this limitation.

### 3.4. Intervention

**Figure 6:**
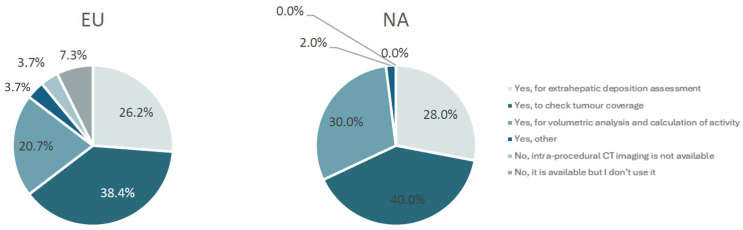
The percentage usage of intra-procedural CT imaging (e.g., cone-beam CT or Angio-CT) by region.

A continental difference was observed in the adoption of intra-procedural CT imaging (cone-beam CT or Angio-CT). Notably, all responding sites in North America utilized this technique, whereas 11% of European sites did not (4% due to accessibility constraints and 7% by preference) (*p* = 0.01) (Figure 6). Although standard catheters predominated in both Europe (90%) and North America (67%), anti-reflux catheters were employed approximately three times more frequently in North America (33%) compared to Europe (9%). Similarly, radial access was utilized approximately three times more often in North America (33%) than in Europe (12%). Nonetheless, femoral access remained the primary approach in general (North America: 65%, Europe: 81%) (*p* < 0.05).

**Figure 7:**
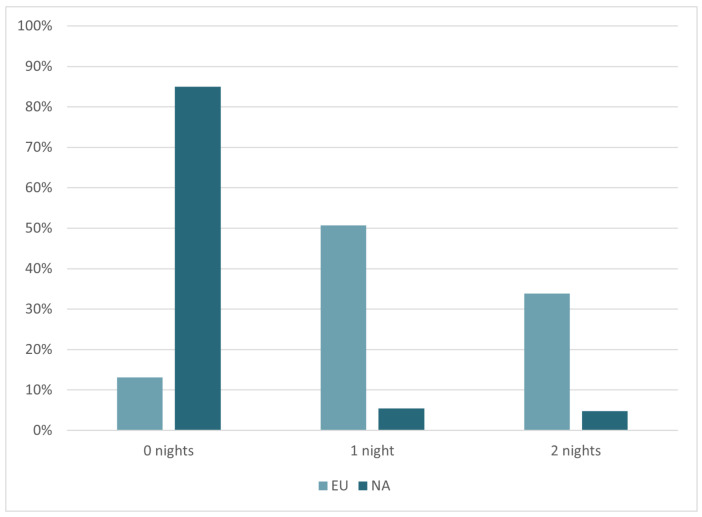
The percentage of patients that remain hospitalised for 0, 1 and ≥2 nights, respectively, by continent.

Radioembolization may be performed as an inpatient procedure or outpatient procedure (i.e., discharged the same day). Outpatient treatments were considerably more frequent in North America (85%) than Europe (13%). In Europe it was most common for patients to stay a minimum of one night (51%) (*p* < 0.05), as shown in Table 5 and Figure 7.

### 3.5. Follow-Up

No significant continental differences were observed in the proportion of centres conducting post-treatment imaging (Europe: 97%, North America: 96%), the preferred imaging modality (SPECT/CT: Europe 40%, North America 45%) (Table 6), or whether centres performed a quantitative evaluation of delivered dose (Europe: 67%, North America: 62%). Complication incidence was low and largely consistent between continents.

### 3.6. Innovations

The majority of responding centres in Europe (80%) and North America (81%) agreed that improved dose calculation methods were a key development to advance radioembolization treatment. Disagreement in the importance of improved catheter designs was noted; approximately twice as many centres in Europe (19%) versus North America (9%) disagreed that improved catheter designs would improve treatment practice.

Innovations highlighted in both continents included combination therapies (e.g., with immuno-oncology) and agents for optimised microsphere distribution (e.g., temporary embolization material). Unique to Europe were calls for ‘updated guidelines to enhance consistency and quality’, while North American centres emphasised ‘faster medical insurance acceptance’ and streamlined processing.

## 4. Discussion

The reported results were distilled from the most comprehensive survey [16,17] on radioembolization to date and provide a robust representation of the European and North American application in its diverse context. The study’s primary objective was to explore the differences in practice across treatment centres in the respective continents. Valuable information on disparities was identified, as well as insights into underlying factors that may have led to this variability. Beyond the superficial correlation with product availability constraints, complex site-specific decision-making processes may be shaped by factors including physician preferences, reimbursement and local clinical experience/outcomes. Consequently, seemingly unremarkable site-level decisions have nuanced connections to broader contextual factors (clinical, technological, etc.), which have wider implications for radioembolization practice as a whole. A global perspective (as was conducted in this work) is therefore essential to reveal these patterns.

The microsphere products utilised in various regions clearly align with product availability issues. Taking ^166^Ho-microspheres as an example, this product was introduced eleven years ago and was only available in Europe; therefore, it had a relatively small user base confined to this region. Comparatively, the ^90^Y microsphere products have been available for more than twenty years and, as a result, ^90^Y resin and ^90^Y glass microspheres account for more procedures than ^166^Ho microspheres.

A continental difference was observed in the length of hospital stay following radioembolization. Outpatient treatments were common in North America, whereas most European patients were hospitalized for at least one night. Correspondingly, radial access (which facilitates earlier mobilisation of patients compared to femoral access) was more prevalent in North America than Europe. The need to deliver cost-effective healthcare is key in both regions; however, emphasis on efficiencies is likely greater in a system driven by insurance structures and reimbursement models that incentivise shorter hospital stays. From another perspective, the practice of keeping patients in hospital for observation (as seen in Europe) may be a precautionary measure to manage complications. The recent literature [18,19] and results of this study have demonstrated that complications are rare and therefore the relevance of routinely keeping patients for an overnight stay may be questioned.

Prophylactic coil embolization, traditionally employed to occlude aberrant vessels and optimize microsphere flow, has seen a decline in practice in recent years. A 2015 study reported that “most experienced centres avoid coil embolization” [20], a sentiment echoed in a 2019 publication [21], which noted that coil embolization is now administered only when strictly necessary. Our survey results show limited use of routine coil embolization across all regions, particularly in North America, where no responding centres reported consistently embolizing any of the considered vessels. Recent clinical trials by high-volume North American centres, such as LEGACY [9] and RASER [10], demonstrate a trend toward more selective treatment approaches. Positive outcomes from these trials have likely influenced local perceptions, driving the adoption of selective treatments and, correspondingly, reducing coil embolization.

Another clear regional difference that was noted was users perceptions of the validity of ^99m^Tc-MAA as a surrogate agent for therapeutic microspheres. While most responders indicated that they believed ^99m^Tc-MAA to be reliable, upon closer examination, it became apparent that users considered it to be valuable for most patients, but not all. The post hoc analysis of the SARAH study provided a crucial insight in this regard: up to 27% of patients showed a poor correlation between ^99m^Tc-MAA and ^90^Y resin distribution. A clear parallel can be drawn between user opinions found in this study and results of the SARAH post hoc analysis [22]; MAA is a good indicator for most patients, but not every patient.

A relatively larger proportion of American users (19%) considered ^99m^Tc-MAA unreliable for intrahepatic dosimetry, compared to only 6% of responders in Europe. Potentially, we are seeing a juncture emerging, and there will be a divergence in practice between the United States and Europe: increased adoption of ablative treatment techniques (where intrahepatic ^99m^Tc MAA-based dosimetry is relatively less important) in the US [10,23], versus dosimetry-led bilobar treatments in Europe [24,25,26]. This divergence may also be artificial, stemming more from the patient populations referred for radioembolization in the respective regions rather than differences in the preferences of interventional radiologists and nuclear medicine teams [27]. Finally, differences may also be attributed to the differing focus of the responding centres, i.e., transplant vs non-transplant. For example, in transplant centres, a greater uptake of ablative style treatment may be expected in order to bridge to transplant or for the purpose of downstaging [28,29,30,31,32].

Despite differences in opinion on the validity of ^99m^Tc-MAA, this has not presented a barrier to routine implementation of personalised dosimetry. The vast majority of users in both Europe (84%) and North America (71%) integrate personalised dosimetry into their treatment planning. Additionally, a potential area of innovation highlighted by users in both regions as was improved dose calculation methods.

Our study has several limitations; to maintain survey feasibility, some potentially informative questions were excluded. For example, the intended vascular treatment or injection level (a determinant of coil embolization) was not captured, which limits interpretation of the coil embolization findings. Additionally, clinical context on treatment intent (i.e., palliative vs curative/ablative) and strategy (i.e., downstaging, radiation lobectomy, or segmentectomy) and their associated dosing goals was not assessed. Future iterations should consider incorporating these categories. The global distribution of responses reveals low participation from certain continents (i.e., Africa and Oceania). This may stem from fewer treatment centres; however, the prominent involvement of European sites was likely influenced by the lead role of the CIRSE. Even within the studied continents, European sites outnumbered North American ones, potentially skewing results and deemphasizing trends from the underrepresented continents. The comparatively lower proportion of high-volume centres in the European vs North American cohorts may also partly shape the observed regional differences. Large differences in procedural volume and therefore experience between institutions likely shape technical decision-making, attitudes toward innovation, and expectations for future developments. For example, guideline updates (which were highlighted in Europe) may hold less immediate relevance for established high-volume programmes. Response bias is a further limiting factor; high-volume centres and research-active institutions are potentially disproportionately represented, since they are more likely to engage with survey initiatives. Consequently, the sample may not be truly representative of the broader radioembolization population. For example, the high proportion of centres routinely conducting quantitative evaluations of post-treatment dose, as identified in this study, appears to be underrepresented in the published literature; this discrepancy may stem from response bias. Trends should be interpreted in this light and caution exercised when extrapolating to infer global patterns.

## 5. Conclusions

This sub-analysis of the CIRSE Global Radioembolization Survey illustrates variability in radioembolization practices across Europe and North America. Key regional differences included hospital stay duration, product usage, and procedural variations. Our findings underscore the need for standardisation efforts, and organizations such as CIRSE will play a crucial role in achieving harmonized global practices.

## Figures and Tables

**Table 1:** Questions and answers as presented in the survey.

Questions	Answers
What is the name of your centre?	Free-text field
Where is your centre located?	City/Country
What year did your centre start performing radioembolization?	Free-text field
How many radioembolization procedures were performed by your centre in the following years?	2017 … 2018 … 2019 … 2020 … 2021 … 2022 (projection) …
How frequently do you encounter the following indications in your centre? (approximate percentage of total patients treated per year)	HCC … Cholangiocarcinoma …Colorectal carcinoma metastasis … Breast liver metastasis … Neuroendocrine liver metastasis … other …
How frequently do you use these microspheres for radioembolization in percent?	^90^Y resin … ^90^Y glass … ^166^Ho …
What is the typical time interval between baseline diagnostic imaging (CT/MRI/other) and work up angiography?	
Do you perform liver function assessment as part of the treatment workup in some, or all of your cases?	Yes, using hepatobiliary scintigraphy (HBS)/Yes, using contrast enhanced MRI with liver specific agents/Yes, other/No
What kind of prophylactic medication do you routinely prescribe pre-, during or post-treatment? Where applicable, please select more than one option.	Steroids/Opioids/NSAIDs/Paracetamol/Metamizole/Anti-emetics/Proton-pump inhibitor/Other
Please indicate how patients are referred in your centre. (MDT = multidisciplinary team meeting)	Through an MDT where an Interventional Radiologist is present/Through an MDT where an Interventional Radiologist is not present/Patients are not referred through an MDT
How many nights do patients normally stay in your hospital for radioembolization? Please estimate the fraction of patients per category.	0 nights/1 night/≥2 nights
What is/are the main reason(s) for you to perform a scintigraphy work-up procedure (^99m^Tc-MAA or ^166^Ho Scout) before radioembolization? Where applicable, please select more than one option.	Lung shunt assessment/Extrahepatic deposition assessment/Intrahepatic dosimetry/Other
What kind of imaging do you use to evaluate the scintigraphy work-up procedure (^99m^Tc-MAA or ^166^Ho Scout)? Where applicable, please select more than one option.	Planar/SPECT/SPECT-CT/Other
Do you consider lung shunting a contraindication to TARE? Please select 0 for both options if you want to indicate that you do not consider lung shunting a contraindication.	Yes, when the shunt is >…%/Yes, when the shunt results in a lung dose > …Gy
How many patients (%) do you exclude due to excessive lung shunt?	
How many patients (%) receive dose reduction due to excessive lung shunting?	
Are patients excluded from treatment if ^99m^Tc MAA or ^166^Ho scout scintigraphy demonstrates poor tumour targeting?	Yes/No
Is personalised dosimetry utilised following ^99m^Tc-MAA or ^166^Ho Scout scintigraphy to determine whether tumour dose will exceed a pre-defined dose threshold?	Yes/No
Do you find ^99m^Tc MAA SPECT/CT reliable for intrahepatic dosimetry?	Yes/No
What method do you use to calculate injected activity for each of the following? ^90^Y resin spheres/^90^Y glass spheres ^166^Ho spheres	BSA/modified BSA/MIRD single compartment/MIRD multi-compartment
Do you use software for dosimetry? Where applicable, please select more than one option.	Yes, MIM Sureplan/Yes, Mirada Simplicit90Y/Yes, Varian RapidSphere/Yes, Terumo QSuite/Yes, Other/No/I do not know/
Which arteries, if any, do you embolise during diagnostic angiography?	Gastroduodenal artery/Right gastric artery/Cystic artery/Other
Do you use intra-procedural CT imaging (e.g., cone-beam CT or Angio-CT) for radioembolization? Where applicable, please select more than one option.	Yes, for extrahepatic deposition assessment/Yes, to check tumour coverage/Yes, for volumetric analysis and calculation of activity/Yes, other/No, intra-procedural CT imaging is not available/No, it is available but I do not use it
What kind of microcatheter do you use for the administration of spheres? Where applicable, please select more than one option.	Standard microcatheter/Anti-reflux microcatheter/Other
In what percentage of cases do you use the following sites for arterial access?	Radial …%/Femoral …%/Other …%
What is your preferred sphere administration technique in case of bilobar manifestation of tumour?	Sequential left—right radioembolization with a time gap/Left and right hepatic artery in a single session/Whole liver (bilobar) infusion in a single session via proper hepatic artery/other
Do you use either the flexdose programme (SIRspheres) or manipulate the calibration date (TheraSphere) to adapt the number of microspheres injected? Where applicable, please select more than one option.	Yes, I primarily use early week 1 TheraSphere/Yes, I primarily use late week 1 TheraSphere/Yes, I primarily use early week 2 TheraSphere/Yes, I primarily use late week 2 TheraSphere/Yes, I primarily use 1 day pre-calibration SIRsphere/Yes, I primarily use 2 days pre-calibration SIRsphere/Yes, I primarily use 3 day pre-calibration SIRsphere/Not applicable/I do not know
Do you use post-treatment imaging to visually evaluate whether the microsphere distribution is as planned? Where applicable, please select more than one option.	Yes, with PET-CT/Yes, with SPECT-CT/Yes, with SPECT/Yes, with ^166^Ho MRI/Yes, with Other/No
Is a quantitative evaluation of post-treatment imaging performed via assessment of absorbed dose?	Yes/No
How frequently (% of all patients) do you encounter complications in radioembolization patients?	Radiation pneumonitis …%/Gastrointestinal complications …%/Pancreatic complications …%/Radioembolization induced liver disease (REILD) …%/Bile duct complications …%/Cholecystitis …%/Abscess …%/Other …%/No complication …%
Which of the following (potential) developments could improve radioembolization treatment in your practice?	New scout agents/Real-time imaging feedback on the dose distribution/Improved dose calculation methods/Improved catheter design/Same day SIRT
Are there any other emerging techniques that you think may improve radioembolization treatment in your practice?	Free-text field

**Table 2:** Responding centres classified by low-, medium-, or high-volume case load.

Region	Low-Volume Centres (%)	Medium-Volume Centres (%)	High-Volume Centres (%)
North America	19% (*n* = 4)	24% (*n* = 5)	57% (*n* = 12)
Europe	34% (*n* = 30)	52% (*n* = 45)	14% (*n* = 12)

Low-volume centres are defined as those performing < 1 radioembolization procedure per month, medium-volume centres are defined as those performing > 1 per month but <1 per week, and high-volume centres are defined as those performing ≥ 1 per week.

**Table 3:** The reported percentage split of total procedures by indications in Europe and North America.

	Indications					
Region	HCC *	mCRC **	ICC ***	Neuroendocrine Metastasis	Breast Cancer Metastasis	Other
Europe	51%	19%	13%	6%	7%	4%
North America	61%	15%	15%	2%	2%	4%

* Hepatocellular carcinoma, ** metastatic colorectal carcinoma, *** Intrahepatic cholangiocarcinoma.

**Table 4:** The reported reliability of ^99m^Tc-MAA for intrahepatic dosimetry in Europe and North America.

	Do You Find ^99m^Tc-MAA Reliable for Intrahepatic Dosimetry?			
Region	Yes, in All Cases	Yes, in Some Cases	Yes, in Few Cases	No
Europe	15%	72%	7%	6%
North America	5%	62%	14%	19%

**Table 5:** The percentage of patients that remain hospitalised for 0, 1 and ≥2 nights, respectively, by continent.

Region	0 Nights (%)	1 Nights (%)	≥2 Nights (%)
North America	85%	5%	5%
Europe	13%	51%	34%

**Table 6:** The modality usage for post-treatment imaging.

Region	SPECT/CT (%)	PET/CT (%)	SPECT (%)	^166^Ho MRI (%)	Other (%)	Not Performed (%)
North America	45%	36%	9%	0%	5%	5%
Europe	40%	38%	9%	7%	3%	3%

## Data Availability

The datasets used and/or analysed during the current study are available from corresponding author on reasonable request.
